# Knowledge, attitudes, and understanding of probiotics among pediatricians in different regions of Saudi Arabia

**DOI:** 10.1186/s12909-021-02499-w

**Published:** 2021-01-21

**Authors:** Mohammed Hasosah, Mansour Qurashi, Abdullah Balkhair, Ziyad Alzahrani, Abdullah Alabbasi, Muhanad Alzahrani, Wejdan Alnahdi, Sohaib Shafei, Malak Bafaqih, Muhammad Khan

**Affiliations:** 1 Pediatric Gastroenterology Department, College of Medicine, King Saud bin Abdulaziz University for Health Sciences, King Abdullah International Medical Research Center (KAIMRC), National Guard Hospital, Jeddah, Kingdom of Saudi Arabia; 2 Neoatology Department, College of Medicine, King Saud bin Abdulaziz University for Health Sciences, King Abdullah International Medical Research Center (KAIMRC), National Guard Hospital, Jeddah, Kingdom of Saudi Arabia; 3 Pediatric Department, College of Medicine, King Saud bin Abdulaziz University for Health Sciences, King Abdullah International Medical Research Center (KAIMRC), National Guard Hospital, PO Box: 8202, Jeddah, 21482 Kingdom of Saudi Arabia

**Keywords:** Probiotics, Pediatricians, Knowledge, Attitudes

## Abstract

**Background:**

Probiotics are live microorganisms that, when administered in adequate amounts, confer a health benefit upon the host. Knowledge and attitudes of health professionals have been reported to be at a medium level for probiotics. The objective was to evaluate the knowledge and practice styles about probiotics among pediatricians working in different regions of Saudi Arabia.

**Methods:**

This cross-sectional study was conducted at pediatric hospitals in Saudi Arabia. A national survey of 550 pediatric providers (PPs) was conducted between January and March 2020 anonymously on their knowledge and practice styles regarding probiotics, and it was completed by pediatric residents (PRs), pediatric specialist (PSs), pediatric consultants (PCs), and pediatric gastroenterologists (PGs).

**Results:**

The survey had a response rate of 82%. Among the respondents, 57.7% were aware of the probiotic’s definition. There were significant differences in the percentage of participants who had little knowledge of probiotics (*P* < 0.05), with the highest being PRs and the lowest being PGs. The most common probiotic used by all participants was *Lactobacillus acidophilus* (63.3%), and *Mycobacterium avium* was prescribed the least often (8.6%). Most PRs and PSs correctly reported that probiotics reduce the risk of antibiotic-induced diarrhea (74.9 and 80.2%, respectively), but there were no significant differences among them.

**Conclusions:**

Significant differences in knowledge and practice patterns exist for probiotics. Identification of knowledge gaps may be useful to develop educational materials to improve the proper definition, knowledge, and use of probiotics.

**Supplementary Information:**

The online version contains supplementary material available at 10.1186/s12909-021-02499-w.

## Background

The International Scientific Association for Probiotics and Prebiotics (ISAPP) published the most recent and widely accepted definition of probiotics as follows: “live microorganisms that, when administered in adequate amounts, confer a health benefit on the host” [1]. Several species of the genera *Bifidobacterium* and *Lactobacillus* claim to have a core benefit on healthy gut microbiota by creating a favorable gut environment [1]. Meta-analyses also suggest that they are effective against infectious diarrhea, antibiotic-associated diarrhea, travelers’ diarrhea, slow gut transit, irritable bowel syndrome, abdominal pain and bloating, and ulcerative colitis [2–4].

Probiotics are considered to be adjunction to conventional therapy along with vitamins, minerals, and other dietary supplements [5].

Today, probiotics are commercially available substances that are found in dietary supplements, drugs, functional foods, and beverages and in products such as skin creams, vaginal capsules, tampons, and chewable tablets for gum health [6]. Despite the widespread and easily accessible evidence that supports the benefits of probiotic use, health professionals may hesitate to recommend probiotics to patients when they receive conflicting messages [6]. Health professionals may have difficulties in processing large volumes of information that are generated by commercial enterprises about the benefits and use of probiotics [7].

Although there is growing global interest in the field, little is known about practicing pediatricians’ perceptions regarding the use and efficacy of probiotics [7]. Information describing how often pediatricians encounter probiotic usage in their practices and their specific recommendations to their patients has not previously been reported. Knowledge of medical care providers’ familiarity and opinions regarding probiotic-based treatments is essential as more patients begin to incorporate these supplements into their medical regimens and as more clinical research investigating probiotic effectiveness becomes available. Sabina et al. surveyed 1066 health professionals and reported that knowledge and attitudes of health professionals were at a medium level of knowledge for probiotics [7]. One of the assessment measures to determine the effectiveness of campaign messages is to conduct knowledge and attitudes surveys of pediatricians. Understanding the spectrum of management styles that are used in the care of children with probiotics would be paramount for improving the quality of care, having a positive effect on a child’s quality of life, and achieving better health outcomes.

This is the first study in Saudi Arabia to directly assess practicing pediatricians’ perceptions on the use and practice patterns for recommending probiotics in the treatment of several disorders. We aimed to investigate the current knowledge, attitude, and practice of pediatricians regarding probiotics in all regions of Saudi Arabia.

## Methods

### Participants

Participants were asked to complete the questionnaire if they were pediatricians. A pediatrician is defined as a physician who is involved in clinical care, research, or teaching related to pediatric medicine. Pediatricians were classified as pediatric residents (PRs), pediatric specialists (PSs), pediatric consultants (PCs), or pediatric gastroenterologists (PGs). PRs are training pediatricians while PSs are certified pediatricians. The study was conducted between January and March 2020.

### Study setting

This cross-sectional national survey was conducted in the following five regions of Saudi Arabia: central region (CR), western region (WR), eastern region (ER), northern region (NR), and southern region (SR). Saudi Arabia’s population is 31 million, and children ages 0 to 14 years represent 29.4% of the population. Pediatricians working in Saudi healthcare system were from university hospitals, governmental hospitals and private hospitals. Probiotics were available over-the-counter (OTC) in Saudi Arabia in a variety of forms such as capsules, packets, or food supplements without a prescription. The survey population consisted of members listed as pediatricians by the Saudi Pediatric Association, the Saudi Commission for Health Specialties, and the Ministry of Health. These criteria resulted in an initial target cohort of approximately 4100 members. From this cohort, a random sample of 550 members was obtained.

### Survey design

We designed a brief, user-friendly questionnaire that assessed the knowledge and attitudes of pediatricians regarding probiotics. The questionnaire was pilot-tested by a sample of PGs. The questionnaire was then revised based on reproducibility, validity, and question value. A group of ten pediatric gastroenterologists was evaluated in the original questionnaire, apart from the final research study (Cronbach’s alpha = 0.8). Reproductively, relevance and query value have been used to update this questionnaire. Changes and modifications were made based on the pilot results. The survey was administered during direct communication (interviewed face-to-face) or via email or telephone. Participants answered the English version of the questionnaire. The survey was estimated to take an average of 10 min to complete.

### Questionnaire instrument

The questions were modeled and changed based on those used in previously published studies on the knowledge of probiotics [8]. The questionnaire consisted of 15 items in the following three subscales: demographics and practice characteristics (five items); definition and knowledge of probiotics (nine items); and source of probiotics-related information (one item). The survey includes multiple-choice questions. All questionnaire items asked participants to choose the best answer. Some response options were on the following scale: all of the time; most of the time; sometimes; seldom (rarely); or never. The datasets used and/or analyzed during the current study available from the corresponding author on reasonable request.

### Questionnaire subscales

#### Demographics and practice characteristics

Participating pediatricians were asked for their age, title, gender, type of practice (general or subspecialty), and level of health care institutions to identify potential differences that may be influenced in the systematic approach to probiotics.

#### Definition, indication, and Management of Probiotics

Participants were asked to provide a definition of probiotics. The respondents were queried as to the most common strain of probiotics. Participants were also surveyed on how to treat infants with probiotics.

#### Source of probiotic-related information

Participants were asked whether they had probiotics-related information available to them. The participants were asked about knowledge of efficacy of probiotics. The participants were asked to rank the best source of probiotics-related information that they used from a list that included medical journals, conferences, newsletters, internet, and pharmaceutical company-sponsored symposia.

### Statistical analysis

Data were analyzed using SPSS PC+ version 21.0 statistical software (SPSS Inc., Chicago, IL, USA). Descriptive statistics (mean, standard deviation [SD], and percentages) were used to describe the quantitative and categorical study and outcome variables. A Pearson’s Chi-square test was used to observe the association between the categorical study and outcome variables. A *P* value of < 0.05 was used to report the statistical significance of results.

## Results

### Demographics

Of the 550 questionnaires that were distributed to participating pediatricians, 452 (response rate, 82%) were completed and analyzed. Thirteen questionnaires were excluded because of missing or incomplete data. Most of the respondents were less than 30 years of age (43.4%), followed by those who were between 30 and 40 years of age (41.4%), those who were between 41 and 50 years old (7.1%), and those who were over 50 years of age (8.2%). Our sample had equal female and male respondents 226 (50.0%). Among them, 44% were PRs, 23.7% were PCs, 19.0% were PSs, and 3.1% were PGs. Respondents from government hospitals represented the highest proportion (89.6%), followed by respondents from private hospitals (10.4%). Demographics and practice characteristics of the study participants are presented in Table 1.

**Table 1:** Demographic and practice characteristics of the study participants

Demographics	n	%
**Age**		
< 30 years	196	43.4
30–40 years	187	41.4
41–50 years	32	7.1
> 50 years	37	8.2
**Gender**		
Male	226	50.0
Female	226	50.0
**Qualification (title /position)**		
PR	199	44.0
PS	86	19.0
ACP	31	6.9
Ass CP	15	3.3
CP	107	23.7
PG	14	3.1
**Region of practice**		
Eastern Region	29	6.4
Western Region	168	37.2
Central Region	174	38.5
Northern Region	53	11.7
Southern Region	28	6.2
**Institution**		
Government hospital	405	89.6
Private hospital	47	10.4
**Total**	**452**	**100**

*PR* Pediatric resident, *PS* Pediatric specialist, *ACP* Assistant consultant of pediatric, *Ass CP* Associate consultant of pediatric, *CP* Consultant of pediatric, *PG* Pediatric gastroenterologist

### Definition, indication, and Management of Probiotics

**Fig. 1:**
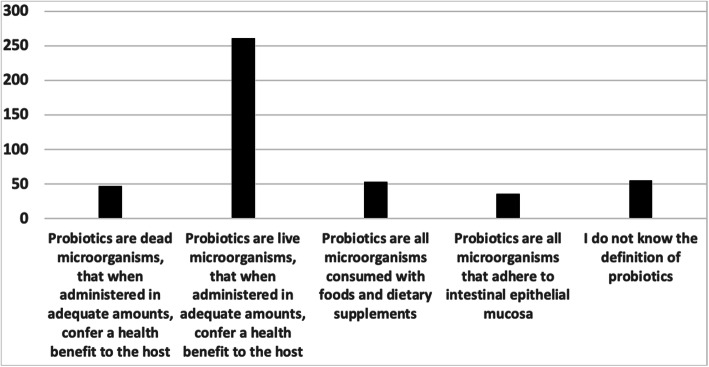
Association between responders’ status and definition of probiotics

When survey respondents were asked about the definition of probiotics, 261 of 452 respondents (57.7%) were aware of the definition of probiotics as live microorganisms, compared with when administered in adequate amounts, confer a health benefit to the host (Fig. 1). Among them, 62.8% of PRs and 48.8% of PSs reported that they have little knowledge of probiotics. However, consultants of pediatrics exhibited little knowledge of probiotics (47.7%), while PGs showed that they have excellent knowledge regarding probiotics (42.9%; *P* < 0.001). Correlation between responders’ status and knowledge about probiotics is shown in Table 2.

**Table 2:** Association between responders’ status and knowledge about probiotics

	How is your knowledge about probiotics?				
**Qualification (title position)**	**No knowledge** **No (%)**	**Little knowledge** **No (%)**	**Medium knowledge** **No (%)**	**Good knowledge** **No (%)**	**P value**
PR	35(17.6)	125(62.8)	37(18.6)	2(1.0)	< 0.001
PS	6(7.0)	42(48.8)	28(32.6)	10(11.6)	
ACP	1(3.2)	14(45.2)	14(45.2)	2(6.5)	
Ass CP	4(26.7)	2(13.3)	7(46.7)	2(13.3)	
CP	1(0.9)	51(47.7)	37(34.6)	18(16.8)	
PG	1(7.1)	3(21.4)	4(28.6)	6(42.9)	
Total No (%)	48(10.6)	237(52.4)	127(28.1)	40 (8.8)	

*PR* Pediatric resident, *PS* Pediatric specialist, *ACP* Assistant consultant of pediatric, *Ass CP* Associate consultant of pediatric, *CP* Consultant of pediatric, *PG* Pediatric gastroenterologist

There were significant differences in the percentage of participants who asked on which systems they think probiotics have an effect. Additionally, 86% of PCs reported that probiotics have effects on the GI system (*P* < 0.001). Assistant consultants (48.4%), associate consultants (40%), PRs (81%), and PSs (68.6%) reported that probiotics have effects on the GI system.

In response to questions about clinical indications for prescribing probiotics, there were no significant differences (*P* = 0.298) between them groups. However, most participants reported that probiotics were used to improve digestion and GI immunity. Correlation between responders’ status and indications of probiotics is shown (Table 3). Among the participants who responded to the survey, most PRs and PSs correctly reported that probiotics reduce the risk of antibiotic-induced diarrhea (74.9 and 80.2%) respectively, but there were no significant differences among them.

**Table 3:** Association between responders’ status and indications of probiotics

	Which of the following systems you think the probiotics has effects?					Why you are prescribing probiotics?					
	GI system No (%)	Immune system No (%)	Respiratory system No (%)	Cardiology system No (%)	p-value	Preventive during antibiotic treatment No (%)	Improved digestion No (%)	Improve GI immunity No (%)	Reduce bloating No (%)	Reduce allergic condition No (%)	p-value
PR	163(81.9)	27(13.6)	6(3.0)	3(1.5)	< 0.001	24(12.1)	84(42.2)	67(33.7)	18(9.0)	6(3.0)	0.298
PS	59(68.6)	18(20.9)	5(5.8)	4(4.7)		15(17.4)	30(34.9)	33(38.4)	6(7.0)	2(2.3)	
ACP	15(48.4)	11(35.5)	3(9.7)	2(6.5)		4(12.9)	8(25.8)	10(32.3)	7(22.6)	2(6.5)	
Ass CP	6(40.0)	5(33.3)	3(20.0)	1(6.7)		2(13.3)	2(13.3)	5(33.3)	4(26.7)	2(13.3)	
CP	92(86.0)	11(10.3)	2(1.9)	2(1.9)		13(12.1)	41(38.3)	42(39.3)	8(7.5)	3(2.8)	
PG	9(64.3)	1(7.1)	2(14.3)	2(14.3)		1(7.1)	3(21.4)	7(50.0)	2(14.3)	1(7.1)	
Total	344	73	21	14		59	168	164	45	16	

*PR* Pediatric resident, *PS* Pediatric specialist, *ACP* Assistant consultant of pediatric, *Ass CP* Associate consultant of pediatric, *CP* Consultant of pediatric, *PG* Pediatric gastroenterologist

When survey respondents were asked about the prescription of probiotics, nearly half of the participants (57.7%) reported that probiotics must be taken before meals. Among them, 61.3% of PRs, 58.9% of PCs, and 57.1% PGs chose the correct answer, but there were no significant differences among them (*P* = 0.182). Association between responders’ status and knowledge of probiotics is shown (Table 4).

**Table 4:** Association between responders’ status and knowledge of probiotics

	Do you think probiotics will reduce the risk of antibiotic-induced diarrhea?			Do you think probiotics should be taken before a meal?			***Lactobacillus rhamnosus*** is the most microbial species in probiotic Strains		
	True No (%)	False No (%)	p-value	True No (%)	False No (%)	p-value	Yes No (%)	No No (%)	p-value
PR	149(74.9)	50(25.1)	0.270	122(61.3)	77(38.7)	0.182	64(32.2)	135(67.8)	0.840
PS	69(80.2)	17(19.8)		40(46.5)	46(53.5)		28(32.6)	58(67.4)	
ACP	18(58.1)	13(41.9)		21(67.7)	10(32.3)		13(41.9)	18(58.1)	
Ass CP	10(66.7)	5(33.3)		7(46.7)	8(53.3)		4(26.7)	11(73.3)	
CP	79(73.8)	28(26.2)		63(58.9)	44(41.1)		35(32.7)	72(67.3)	
PG	10(71.4)	4(28.6)		8(57.1)	6(42.9)		6(42.9)	8(57.1)	
Total	335	117		261	191		150	302	

*PR* Pediatric resident, *PS* Pediatric specialist, *ACP* Assistant consultant of pediatric, *Ass CP* Associate consultant of pediatric, *CP* Consultant of pediatric, *PG* Pediatric gastroenterologist

**Fig. 2:**
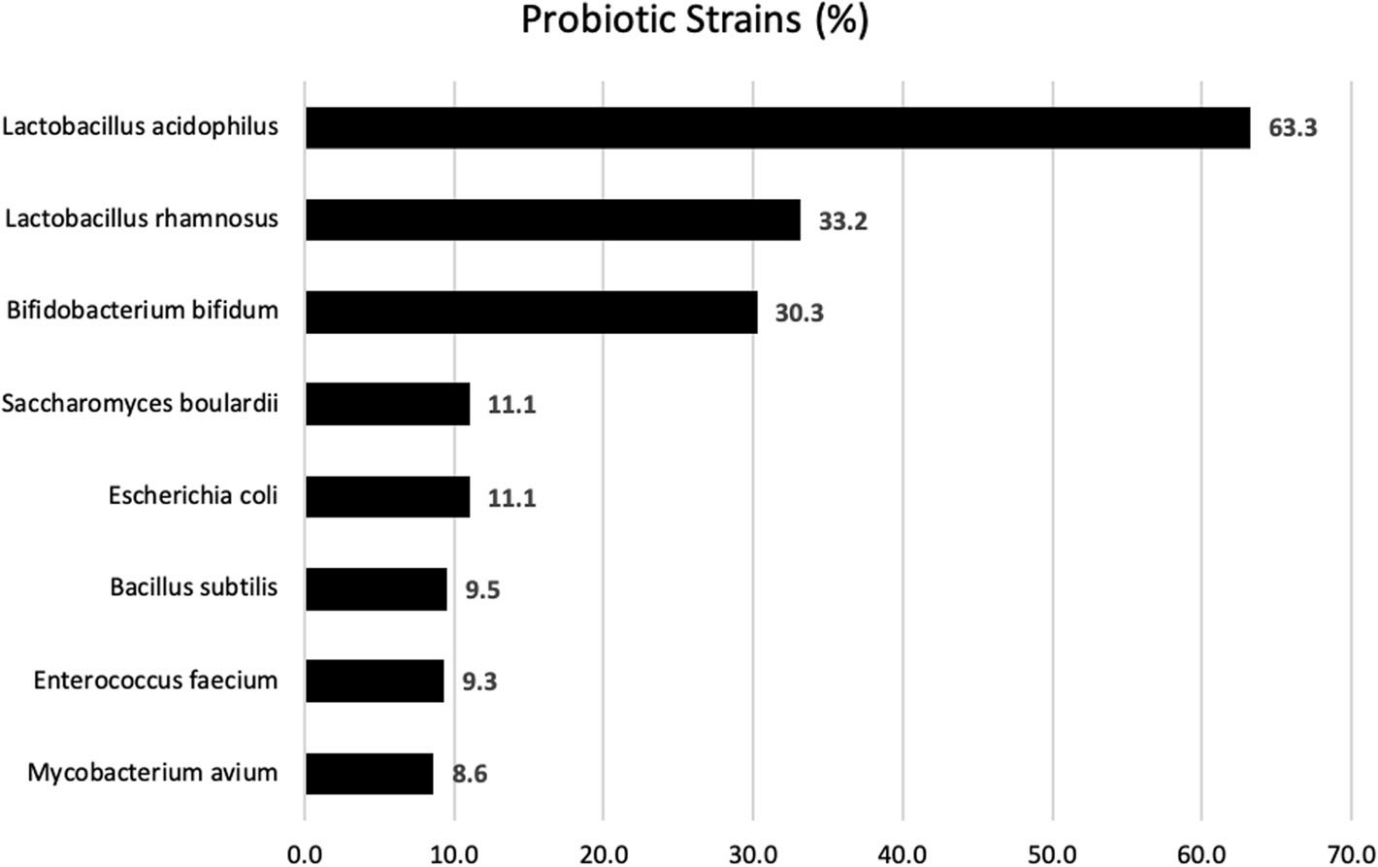
Lists of common probiotic strains prescribed

The most common probiotic used by all participants was *Lactobacillus acidophilus* (63.3%), and *Mycobacterium avium* was the probiotic least often prescribed (8.6%). The lists of common probiotic strains prescribed were shown (Fig. 2). There was no significant difference in *Lactobacillus rhamnosus* as probiotic strain that was used by pediatricians (*P* = 0.840). Correlation between the responders’ status and knowledge of probiotics is shown (Tables 3 and 4).

Regarding the source of probiotics-related information, our study showed that the sources of pediatricians’ information on probiotics included the internet (50.0%), medical journals (44.0%), medical conferences (28.8%), newsletters (11.9%), and radio or TV (8.0%).

In summary, an unanticipated finding of this study was the possible differences in probiotic use practice patterns among PS, PR, PG, ACP, and CP. Overall, the responses indicated that PR and CP were more likely to report themselves as familiar with the literature related to probiotics and the various available probiotic preparations.

## Discussion

To the best of our knowledge, this was the largest study in the Middle East describing pediatricians’ knowledge, attitude, and practice regarding probiotics. Stanczak et al. surveyed 335 primary care physicians and reported that 38.5% of respondents had heard of probiotics, but only 27.2% stated that they knew what probiotics are [9]. The present study reflects that 57.7% of respondents were aware of the definition of probiotics.

In our study, it was discouraging to find that more than half of the pediatricians reported that they had little knowledge of probiotics, and significant differences in knowledge were noted between PCs from other pediatricians. These data may be explained by a lack of educational materials and different access to resources in some regions of Saudi Arabia. Together with our observation, the data demonstrated that probiotics are popular among gastroenterologists for the treatment of gastrointestinal disorders [10].

In the present study, *L. acidophilus* (63.3%) was the most common probiotic used by all participants. However, Draper et al. demonstrated that *Lactobacillus* GG was often prescribed for general bowel health [11]. Another study showed that most surveyed physicians prescribed *Bifidobacterium infantis* and VSL#3 frequently for irritable bowel syndrome and antibiotic-associated diarrhea [12]. The probiotic strains belonging to the *Lactobacillus* and *Bifidobacterium* genera that are most commonly used as probiotics are well known in the literature [13, 14]. Our observation in the present data showed that probiotic prescribing is common, but it lacks consistency, with the choice of probiotic frequently left to the patient, even for indications with some strain-specific evidence. These different kinds of probiotics that were prescribed by participants may suggest using various educational tools, including peer-reviewed publications, media, seminars, and university courses to introduce the concepts and explain indications, advantages, and limitations of these probiotics.

Most of our respondents (86%) reported that probiotics were used to improve digestion and improve gastrointestinal immunity. Similar to Williams et al. [12] 98% of the respondents in our study believed that probiotics have a role in treating gastrointestinal illnesses or symptoms [12].

Thus, our participants’ observations suggest that common indications for probiotics were prevention and treatment of antibiotic-associated diarrhea [11, 12]. However, many PGs worldwide do not use probiotics for acute infectious diarrhea because of a lack of appropriate guidelines and/or poorly designed products [15]. Meta-analyses have shown probiotics are well documented, and their use alone or in combination with other therapies can, therefore, be considered “evidence-based,” such as for antibiotic-associated diarrhea in adults and children [16].

Several systematic reviews on adult and pediatric antibiotic-associated diarrhea (AAD) suggest that probiotic bacteria offer a solution. Data indicate that Lactobacillus strains in particular seem to be effective. The latest meta-analysis of 10 randomized control trials testing the efficacy of S. boulardii in preventing AAD shows an overall, pooled relative risk of 0.47 {95% confidence interval (CI) = 0.35, 0.63; *p* < 0.001} [16].

In response to the question of how respondents prescribed probiotics, most (58%) reported that probiotics should be taken before a meal with no significant difference *P*-value among pediatricians. Similar to the observations by Tompkins et al., Sabina et al. confirmed the highest survival of probiotics if given with a meal or before a meal, and the lowest survival if taken after a meal [7, 17]. These results emphasize the importance for healthcare professionals to be properly educated and updated on probiotics because improved knowledge about probiotics would lead to increased prescriptive confidence [16].

The strengths of our study are that the study is cross-sectional, the survey was conducted in all regions of Saudi Arabia, and most questionnaires were filled out under the supervision of the investigators to avoid misinterpretation of the questions. This study has several limitations. There may be some response bias—PPs who have a special interest in probiotics may have been more likely to respond to the survey. Thus, the state of knowledge of probiotics among PPs may be even lower than that reported in this study. The PR and PCs in our sample demonstrated a higher response rate than others, possibly because of more interest or more exposure to probiotics-related disorders.

In summary, our study contributed to a better understanding of probiotics in the clinical practice of Saudi pediatricians who are involved in pediatric healthcare. Effective implementation of this practice will benefit from additional supporting studies and the eventual development of clinical practice guidelines that are supported by the Saudi Gastroenterology Society.

## Conclusion

This study provides valuable insight into the knowledge and practice of pediatricians working in Saudi Arabia regarding probiotics. There are significant differences in the knowledge gap and practice patterns exist among pediatricians from different regions of Saudi Arabia regarding the definition, knowledge, and use of probiotics. Identification of gaps in knowledge and practice may be helpful to policymakers who are in charge of developing educational materials for pediatricians about providing knowledge on probiotics.

## Supplementary Information

**Additional file 1.** Questionnaire.

## Data Availability

Not applicable.

## Abbreviations

PPs: Pediatric providers
PRs: Pediatric residents
PSs: Pediatric specialists
PCs: Pediatric consultants
PGs: Pediatric gastroenterologists
ISAPP: International Scientific Association for Probiotics and Prebiotics
