# Supplementary material for: Knowledge, attitudes, and understanding of probiotics among pediatricians in different regions of Saudi Arabia
Source: BMC Med Educ. 2021 Jan 21;21:68. doi: 10.1186/s12909-021-02499-w (PMC7819255; doi:10.1186/s12909-021-02499-w)
Supplement: Supplementary file 1 — Additional file 1. Questionnaire. [file 12909_2021_2499_MOESM1_ESM.docx]

**Questionnaire**

**Knowledge, Attitudes to and Understanding of Probiotics Among Pediatricians in Western Region, Saudi Arabia**

--------------------------------------------------------------------------------------------------------------------

Dear Doctor,

Thank you for your participation in this survey “Knowledge, Attitudes to and Understanding of Probiotics Among Pediatricians in Western Region, Saudi Arabia”

Our objective from this survey is to evaluate the current knowledge, attitude, and practice of pediatricians regarding probiotics.

1. **Age:**

- < 30-year-old
- 31-40-year-old
- 41-50-year-old
- >50-year-old

1. **Gender:**

- Male
- Female

1. **Qualification (title position):**

- Pediatric resident
- Pediatric specialist
- Junior pediatric consultant
- Senior pediatric consultant
- Pediatric gastroenterologist

1. **Where is your region of practice?**

- Jeddah
- Makkah
- Taif

1. **Institution:**

- Government hospital
- Private hospital
- Others: ………………

1. **How is your knowledge about probiotics?**

- No knowledge
- Little knowledge
- Medium knowledge
- Good knowledge

1. **In your opinion, what is the definition of probiotics?**

- probiotics are dead microorganisms, that when administered in adequate amounts, confer a health benefit to the host
- probiotics are live microorganisms, that when administered in adequate amounts, confer a health benefit to the host
- probiotics are all microorganisms consumed with foods and dietary supplements
- probiotics are all microorganisms that adhere to intestinal epithelial mucosa
- I do not know the definition of probiotics.

1. **Which microbial species you would believe that Probiotic Strains?**

- Lactobacillus acidophilus
- Bifidobacterium bifidum
- Mycobacterium avium
- Escherichia coli
- Lactobacillus rhamnosus
- Bacillus subtilis
- Enterococcus faecium
- Saccharomyces boulardii

1. **Which of the following systems you think the probiotics has effects?**

- GI system
- Immune system
- Respiratory system
- Cardiology system

1. **In your opinion, why you are prescribing probiotics?**

- Preventive purposes during an antibiotic treatment
- Improved digestion
- Improve GI immunity
- Reduce bloating
- Reduce allergic conditions

1. **Which kind of probiotics form you like to prefer?**

- Tablets
- Powders
- Capsule
- Syrup/drops

1. **Do you think probiotics will reduce the risk of antibiotic-induced diarrhea?**

- True
- False

1. **Do you think probiotics should be taken before a meal?**

- True
- False

1. **Do you think that probiotics are harmful for health?**

- True
- False

1. **What are the Sources of Information on** **Probiotics?**

- Medical journals
- Conferences
- Newsletters
- Internet websites
- Radio or TV
- Other, Specify:

******************************************************************************

**Thank you for your participation in this survey**
